# 1260. A Retrospective Observational Study on Assessing the Use of Antibiotic Prophylaxis for Outpatients in the Dental and Ophthalmic Department in a Tertiary Care Hospital

**DOI:** 10.1093/ofid/ofad500.1100

**Published:** 2023-11-27

**Authors:** Suresh Kumar Dorairajan, Ratheesh Rajakumar, Safiya Banu Sadik Basha

**Affiliations:** Apollo Hospitals, Chennai, Tamil Nadu, India; Jaya College of Paramedical Sciences, chennai, Tamil Nadu, India; Jaya College of Paramedical Sciences, chennai, Tamil Nadu, India

## Abstract

**Background:**

Antibiotics are most commonly used in outpatient setting when compared to inpatient setting. Inappropriate antibiotics prescribing in dental and ophthalmic outpatient setting without following any proper guidelines has been identified as a potential driver for antimicrobial resistance especially in the developing world. As there is no sufficient data in India regarding efficient prophylactic use of antibiotics in dental and ophthalmic department, this study is aimed to address this by assessing the antibiotic prophylaxis among outpatients.

**Methods:**

This retrospective observational study was conducted in the Dental and Ophthalmic outpatient’s department in a tertiary care hospital in South India for 3 months from April 2022 to June 2022. The data regarding the Antibiotic prophylaxis were collected from records of the patients’ prescription and analyzed using simple statistics.

**Results:**

A total of 395 prescriptions are collected. Mean age was found to be 53.5 years. The key findings are summarized in Table 1 below.

**Conclusion:**

85% of patients were prescribed with antibiotics in Dental OP whereas 90% of patients were prescribed with antibiotics in Ophthalmic OP in which 56% of patients were found to have inappropriate duration of Antibiotic therapy. Understanding the current patterns of antibiotic prophylaxis in outpatient setting is necessary to implement antibiotic stewardship and large multicentric studies in the future will help to reduce unnecessary antibiotic use in the developing world.

**Disclosures:**

**All Authors**: No reported disclosures

**Table 1.** Baseline characteristics of Outpatients in Dental and Ophthalmic Department

Departments		Dental	Ophthalmic
Total Patients		150	245
Total Patients	Male	78(52%)	129 (52.7%)
	Female	72(48%)	116 (47.3%)
Antibiotic Prophylaxis	Yes	88(58.7%)	221 (90.2%)
	No	62(41.3%)	24 (9.8%)
Antibiotic Therapy	Monotherapy	85(96.6%)	211(95.5%)
	Combination Therapy	3(3.4%)	10(4.5%)
Duration of Antibiotic Therapy	1-10 days	88(100%)	63(28.5%)
	11-20 days		114(51.5%)
	1 month		30(13.6%)
	1½ months		1(0.5%)
	2 months		12(5.4%)
	4 months		1(0.5%)

